# Berries and Their Polyphenols as a Potential Therapy for Coronary Microvascular Dysfunction: A Mini-Review

**DOI:** 10.3390/ijms22073373

**Published:** 2021-03-25

**Authors:** Rami S. Najjar, Arielle M. Schwartz, Brett J. Wong, Puja K. Mehta, Rafaela G. Feresin

**Affiliations:** 1 Department of Nutrition, Georgia State University, Atlanta, GA 30302, USA; rnajjar1@student.gsu.edu; 2 J. Willis Hurst Internal Medicine Residency Program, Emory University, Atlanta, GA 30322, USA; arielle.m.schwartz@emory.edu; 3 Department of Kinesiology & Health, Georgia State University, Atlanta, GA 30302, USA; bwong@gsu.edu; 4 Division of Cardiology, Emory Women’s Heart Center, Emory University School of Medicine, Atlanta, GA 30322, USA; 5 Division of Cardiology, Emory Clinical Cardiovascular Research Institute, Emory University School of Medicine, Atlanta, GA 30322, USA

**Keywords:** ischemic heart disease, endothelial dysfunction, microvascular, berries, polyphenols, inflammation, oxidative stress, angiotensin, adrenergic

## Abstract

Ischemia with no obstructive coronary artery disease (INOCA) is a common diagnosis with a higher prevalence in women compared to men. Despite the absence of obstructive coronary artery disease and no structural heart disease, INOCA is associated with major adverse cardiovascular outcomes as well a significant contributor to angina and related disability. A major feature of INOCA is coronary microvascular dysfunction (CMD), which can be detected by non-invasive imaging and invasive coronary physiology assessments in humans. CMD is associated with epicardial endothelial-dependent and -independent dysfunction, diffuse atherosclerosis, and left-ventricular hypertrophy, all of which lead to insufficient blood flow to the myocardium. Inflammatory and oxidative stress signaling, upregulation of the renin-angiotensin-aldosterone system and adrenergic receptor signaling are major drivers of CMD. Treatment of CMD centers around addressing cardiovascular risk factors; however, there are limited treatment options for those who do not respond to traditional anti-anginal therapies. In this review, we highlight the ability of berry-derived polyphenols to modulate those pathways. The evidence supports the need for future clinical trials to investigate the effectiveness of berries and their polyphenols in the treatment of CMD in INOCA patients.

## 1. Introduction

A large proportion of patients with signs and symptoms of myocardial ischemia, do not have obstructive coronary artery disease (CAD) on coronary angiography [1,2,3]. Over the last 20 years, ischemia with no obstructive coronary artery disease (INOCA) has been increasingly recognized for its role in contributing to adverse cardiovascular outcomes [1,2,4]. Approximately two-thirds of women who undergo coronary angiography have INOCA [3]. Despite the absence of an arterial blockage, the morbidity and mortality in patients with INOCA is high and includes cardiovascular death, myocardial infarction, stroke, heart failure [3,5]. Indeed, women with INOCA are four times more likely to be re-hospitalized within 180 days of discharge compared to men [3]. A major driver of INOCA is coronary microvascular dysfunction (CMD) [6]. In CMD, the microvasculature is unable to meet myocardial oxygen demands, despite having a structurally normal heart and normal appearing epicardial coronary arteries [7]. In this review, we highlight the pathophysiologic mechanisms contributing to CMD in patients with INOCA. We also discuss the therapeutic potential of berries and their polyphenols in the management of CMD in this patient population. 

## 2. Coronary Microvascular Dysfunction

CMD can be detected by invasive coronary functional angiography or by non-invasive imaging modalities [8]. Invasive techniques include measuring coronary flow reserve (CFR) by a doppler flow wire in the epicardial coronary arteries. Flow velocity changes are measured in response to adenosine. CFR is a ratio of coronary flow velocity at peak hyperemia to resting flow velocity, and a CFR < 2.5 is diagnostic of CMD [8,9]. Abnormal myocardial flow reserve can also be detected non-invasively by cardiac positron emission tomography (PET) imaging, which enables global myocardial blood flow quantification at rest versus stress, in addition to flow reserve in individual coronary territories [10]. CMD can also be detected by stress cardiac magnetic resonance imaging at specialized centers. To test endothelial-dependent epicardial and microvascular function, intracoronary acetylcholine is used during invasive functional coronary angiography [11]. While a normal response to acetylcholine is vasodilation, in patients with underlying endothelial dysfunction or atherosclerosis, there is paradoxical vasoconstriction with acetylcholine in patients with CMD [12]. Women seem to be more susceptible to abnormal coronary vasoreactivity, and both abnormal responses to adenosine and acetylcholine are associated with adverse outcomes in women [8].

Pre-arteriole (100–400 μm) and arteriole (40–100 μm) vessels of the epicardium characterize the primary sites of vascular resistance in cardiac microcirculation, representing 20% and 60% of total resistance, respectively [7]. Myocardial oxygen needs are dependent upon this microcirculatory network [13]. Thus, pathological perturbations, as seen in CMD, can result in ischemic conditions. CMD can occur in the presence of obstructive CAD, and also occurs due to myocardial causes such as hypertrophic obstructive cardiomyopathy, infiltrative or dilated cardiomyopathy, or pressure-induced left ventricular hypertrophy (LVH) [14,15]. However, in INOCA patients, CMD is present despite overt structural heart disease. One should note that in a majority of patients with CMD, diffuse coronary atherosclerosis is present (detected by intravascular ultrasound) [16,17]. Both endothelium-dependent and -independent mechanisms contribute to abnormal myocardial blood flow regulation in CMD [18]. When these pathways are impaired, the microvasculature is not able to augment flow appropriately, creating supply–demand mismatch, which is manifested as ischemia and angina [19]. 

It is interesting to note that women who present with ischemic heart disease are far less likely to have focal atherosclerotic obstruction and more likely to have diffuse atherosclerosis, which partially explains the observed sex-differences in INOCA morbidity [20]. This difference in phenotype may be due to smaller epicardial arteries in women [21] coupled with significantly higher coronary blood flow at both rest and during stress [22] facilitating conditions of inherently higher shear stress. Under atherosclerotic conditions, higher shear stress facilitates diffuse atherosclerosis compared with lower shear stress conditions, which are more conducive to focal atherosclerosis [23]. 

## 3. Mechanisms of CMD 

Mechanisms underlying CMD are not fully understood [24,25]. Ischemic heart disease risk factors such as hypertension, diabetes, and estrogen deficiency have all been implicated [26,27,28]; however, CMD is poorly predicted by traditional risk factors [29].

### 3.1. Oxidative Stress and Inflammation

Underlying inflammation and oxidative stress are key pathophysiologic mechanisms implicated in CMD as both oxidative stress and inflammation are known to impair vascular endothelial function [30,31,32,33]. Increased vascular oxidative stress, characterized by an increase in reactive oxygen species (ROS), is caused by an imbalance between ROS-producing and -degrading enzyme systems. An increase in ROS levels can lead to thiol oxidation, which has been shown to be higher in ischemic heart disease patients [34,35]. Further, increased ROS can contribute to activation of redox-dependent inflammatory mediators exacerbating inflammation. Patients with CMD express higher systemic concentrations of tumor necrosis factor (TNF)-α [36,37]. In fact, TNF-α inhibition in patients with CMD significantly improves CFR [38]. Further, in patients with non-obstructive coronary artery disease, high sensitivity C-reactive protein (hs-CRP) correlates with both angina as well as markers of ischemia [39]. 

### 3.2. Endothelial-Dependent and -Independent Dysfunction

Endothelial cell (EC)- and vascular smooth muscle cell (VSMC)-derived oxidative stress are primarily mediated via NADPH oxidases (NOX), which abstract electrons from NAPH to O_2_ to yield ROS, i.e., superoxide (O_2_^•−^) and hydrogen peroxide (H_2_O_2_) [40]. Significant crosstalk occurs between the endothelium and VSMCs. ROS facilitates endothelial dysfunction via diminished production and bioavailability of nitric oxide (NO), a potent vasodilator. NO facilitates vasodilation in VSMCs via interaction with soluble guanylate cyclase (sGC) and a subsequent increase in cGMP, cGMP kinases and a consequent decrease in intracellular Ca^2+^, decreasing myosin-dependent contraction [41]. ROS antagonizes NO signaling by increasing VSMC intracellular Ca^2+^ while also directly interacting with NO to form peroxynitrite (ONOO^−^), a reactive nitrogen species (RNS) [42]. Further, ROS facilitates uncoupling of endothelial NO synthase (eNOS) due to impaired GTP-cyclohydrolase activity and, subsequently, reduced tetrahydrobiopterin (BH_4_) synthesis, a key substrate for eNOS [43]. With reduced BH_4_ bioavailability, eNOS produces O_2_^•−^ at the expense of NO. NOX facilitates endothelial-independent dysfunction in VSMCs by increasing sarcoplasmic release of Ca^2+^ [44]. Thus, oxidative stress affects both endothelial-dependent and -independent pathways.

**Figure 1:**
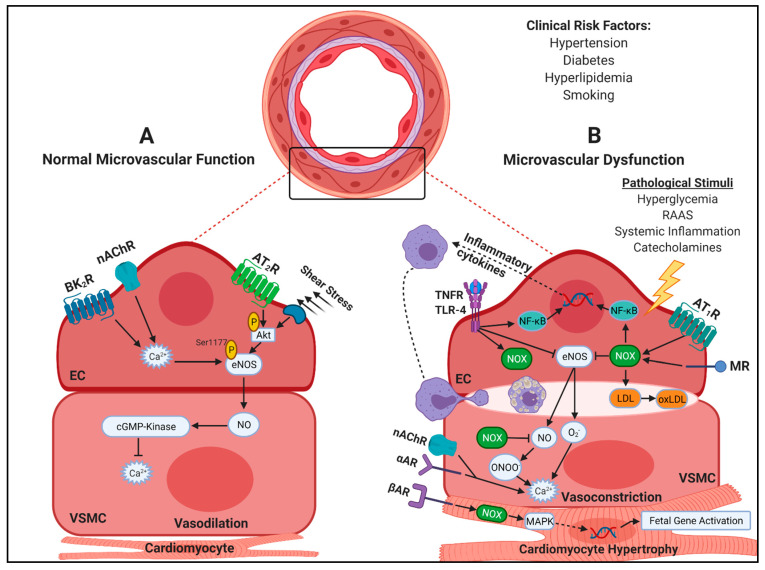
Cellular Mechanisms Contributing to Coronary Microvascular Dysfunction (CMD). In the microvessels of the epicardium, endothelial function is regulated by interactions between endothelial cells (ECs) and vascular smooth muscle cells (VSMCs). (**A**) In normal endothelial function, bradykinin receptor B2 (BK_2_) and nicotinic acetylcholine receptor (nAChR) activation triggers Ca^2+^-dependent endothelial nitric oxide synthase (eNOS) activation. Additionally, angiotensin II type 2 receptor (AT_2_R) activation, as well as detection of shear stress from cell membrane caveolae, lead to phosphorylation of protein kinase B (Akt) and subsequent phosphorylation of eNOS at Ser1177. Nitric oxide (NO) produced from eNOS diffuses into VSMCs, leading to eventual cGMP-kinase activation and sarcoplasmic reticulum Ca^2+^ uptake, allowing vasodilation. (**B**) Clinical risk factors of CMD include hypertension, diabetes, hyperlipidemia and smoking. Microvascular dysfunction is induced by hyperglycemia, components of the renin-angiotensin-aldosterone system (RAAS) as well as catecholamines and inflammatory cytokines. These stimuli have selectivity for a number of receptors, including angiotensin II type 1 receptor (AT_1_R), tumor necrosis factor receptor (TNFR), Toll-like receptor (TLR)-4, mineralocorticoid receptor (MR), and β-adrenergic receptor (βAR), which induce NADPH-oxidase (NOX) activation as well as nuclear translocation of nuclear factor kappa-light-chain-enhancer of activated B cells (NF-κB). Nox activation and ROS production exacerbates the cellular inflammatory response by enhancing NF-κB activation via upstream redox sensitive kinases. Upon NF-κB nuclear translocation, inflammatory cytokines and chemokines are expressed, facilitating endothelium permeability and macrophage recruitment and infiltration. Trapped low-density lipoproteins (LDL) are oxidized by NOX and ingested by macrophages leading to foam cell formation in the sub-endothelial space and diffuse atherosclerosis, impeding normal vascular tone. In INOCA diffuse atherosclerosis is present, despite no obstructive stenotic lesion. TNFR activation decreases eNOS expression, while NOX facilitates eNOS uncoupling, leading to superoxide (O_2_^•−^) synthesis at the expense of NO. Further, ROS produced from NOX interacts with NO to form peroxynitrite (ONOO^−^). Reduced NO prevents cGMP-kinase activation in VSMCs and increased ROS; α-adrenergic receptor (αAR) and nAChR activation in VSMCs facilitates aberrant intracellular Ca^2+^ fluctuations leading to vasoconstriction. Excessive ROS produced by NOX facilitates mitogen activated protein kinase (MAPK) signaling in cardiomyocytes, leading to fetal gene transcription of β-myosin heavy chain, α-skeletal muscle and α-smooth muscle actin causing cellular hypertrophy and narrowing of pre-arteriole and arteriole luminal space. Created with Biorender.com, accessed on 24 February 2021.

Further exacerbating microvascular dysfunction, inflammatory cytokines, such as TNF-α, can downregulate eNOS and activate NOX independently [45]. Interestingly, acetylcholine acts as a vasodilator in healthy vessels in an endothelial-dependent manner by increasing NO. However, in endothelial dysfunction, acetylcholine cannot act on ECs due to impaired eNOS and reduced NO, and instead acts on VSMCs facilitating intracellular Ca^2+^ flux and subsequent vasoconstriction [11]. Thus, oxidative stress and inflammation play important roles in mediating endothelial function (Figure 1).

### 3.3. Pathological Remodeling

Both diffuse atherosclerosis of the epicardial microvasculature and LVH severely impede arteriole and pre-arteriole diameter by means of reduced luminal space and abnormal vessel architecture, which results in increased vascular resistance [46]. Atherosclerosis and LVH are driven by inflammation and oxidative stress, with NOX enzymes playing a central role. In atherosclerosis, ROS can trigger downstream activation of nuclear factor kappa-light-chain-enhancer of activated B cells (NF-κB), an inflammatory nuclear transcription factor [47]. Upon NF-κB phosphorylation and subsequent binding to the IκB region of DNA, inflammatory cytokines and adhesion molecules are expressed on the endothelium, leading to leukocyte recruitment and infiltration into the sub-endothelial space [48]. Further, circulating low-density lipoproteins (LDL) become trapped in the sub-endothelium in these pathological conditions and undergoes oxidation by NOX, leading to macrophage ingestion and foam cell formation [49]. In LVH, inflammatory signaling via mitogen activated protein kinases (MAPK) is activated upstream by ROS and facilitates the transcription of cardiomyocyte fetal genes: β-myosin heavy chain, α-skeletal muscle and α-smooth muscle actin. This increases cardiomyocyte cross-sectional area leading to LVH [50]. Thus, targeting inflammatory and oxidative stress pathways in CMD is of major therapeutic significance in both functional and structural aspects (Figure 1).

### 3.4. Renin-Angiotensin System

**Figure 2:**
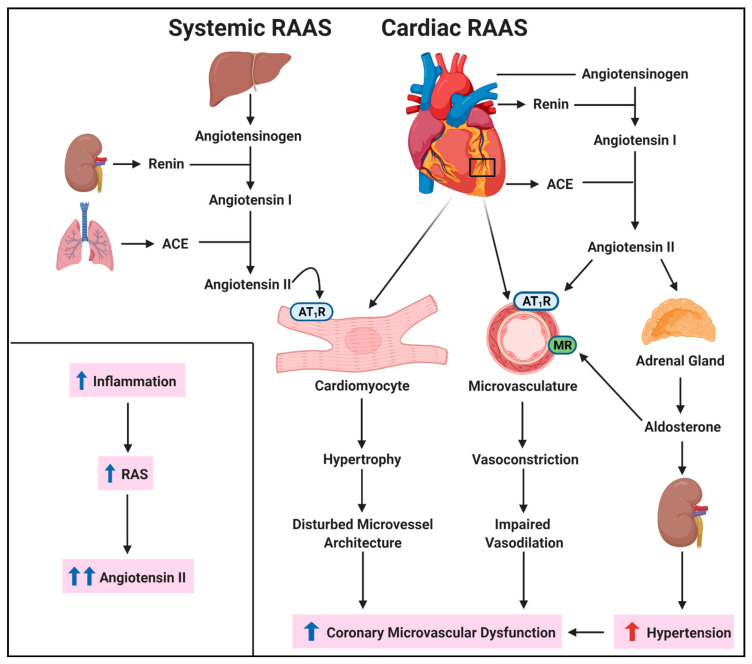
Renin-angiotensin-aldosterone system (RAAS) in CMD. The RAAS is upregulated under inflammatory conditions, leading to upregulated synthesis of angiotensin (Ang) II. Ang II production can occur both systemically and at the organ level and can affect the epicardial microvasculature by promoting vessel vasoconstriction and cardiomyocyte hypertrophy via the Ang II type 1 receptor (AT_1_R). Additionally, aldosterone increases sodium resorption in the kidneys, promoting increased blood volume and high blood pressure. Aldosterone also increases mineralocorticoid receptor (MR) activity on the endothelium, exacerbating endothelial dysfunction. This increases microcirculatory resistance. Cumulatively, these effects result in coronary microvascular dysfunction. Created with Biorender.com, accessed on 24 February 2021.

The renin-angiotensin-aldosterone system (RAAS) is a primary mediator of blood pressure and fluid homeostasis [51]. Classically under conditions of low blood pressure, angiotensinogen (AGT) is produced by the liver and is cleaved by renin, produced by the kidneys to yield angiotensin (Ang) I. Angiotensin converting enzyme (ACE) is produced by lung and further cleaves Ang I to produce Ang II. Ang II is a ligand for angiotensin II type 1 and type 2 receptors (AT_1_R and AT_2_R, respectively) which have differing effects on various tissue. While Ang II-AT_1_R binding typically elicits a pathological response, Ang II-AT_2_R biding mitigates these effects via negative feedback [52]. AT_1_R signaling in the proximal tubules of the kidneys increases Na^+^-H^+^ exchange, which increases the osmolarity of the blood, leading to increased blood volume [52]. Kidneys also respond to aldosterone and antidiuretic hormone (ADH), which are secreted by the adrenal cortex and posterior pituitary, respectively, in response to Ang II signaling [53,54]. Aldosterone secretion increases sodium reabsorption, while ADH promotes fluid resorption, increasing blood volume leading to increased blood pressure. In VSMCs, AT_1_R signaling promotes vasoconstriction, whereas in ECs, this is potentiated by increased NOX expression, reducing NO bioavailability due to direct NO interaction with ROS forming ONOO^−^, an RNS [55] (Figure 1). However, excessive RAAS activation due to inter-organ low-grade inflammation [56,57] can cause hypertension, endothelial dysfunction, cardiac oxidative stress, and hypertrophy, all of which drive the pathogenesis of CMD (Figure 2). Indeed, targeting RAAS with ACE inhibitors improve CFR in women with INOCA [58] and regresses arteriolar wall hypertrophy in hypertension-induced cardiovascular disease (CVD) [59]. Increased blood pressure in hypertension increases microvascular resistance, further exacerbating blood flow within the microcirculatory network [60]. Thus, targeting RAAS is a major therapeutic target.

### 3.5. Adrenergic Receptors in CMD

A major therapeutic target in CVD are the adrenergic receptors. β-adrenergic receptor (βAR) is a G-protein coupled receptor with isoform 1 (β1AR) being expressed primarily in the heart, while isoform 2 (β2AR) is found throughout the vasculature and the heart [61]. The α-adrenergic receptor (αAR) is expressed to a lesser extent in the heart but is highly expressed in the vasculature [62] with isoform 1 (α1AR) being the predominant isoform of clinical interest. Adrenergic receptors are overstimulated by epinephrine and norepinephrine during times of decreased cardiac output, a compensatory mechanism to increase cardiomyocyte excitation and contraction primarily via β1AR [63] as well as an increase in blood pressure via α1AR-mediated VSMC contraction [64] (Figure 1). Indeed, a subset of patients with INOCA may have abnormal cardiac sympathetic activity [65,66] and in the context of CMD, β1AR blockers such as carvedilol improve CFR [67]. These effects may be attributed to partial co-inhibition of αARs, leading to improvements in vasodilation [68]. While αAR blockers do reduce BP [69], their use in heart failure (HF) has led to a worsening of HF and increased mortality [70]. β1AR blockers can improve ejection fraction, reduce arrhythmias, and reduce mortality from HF [63]. Cardiac β1AR overstimulation can cause cardiomyocyte toxicity, and transgenic animal models in which β1AR is increased 15-fold has exhibited progressive decline in heart function, reducing ejection fraction to 20% causing overt HF [71]. 

## 4. Therapeutics in CMD

Treatment of symptomatic patients with CMD can be challenging and therapeutic strategies are under-developed. Management revolves around using anti-anginal, anti-ischemic, and anti-atherosclerotic medications, although large randomized controlled trials are needed [7]. Treatment of modifiable cardiac risk factors such as hypertension and diabetes are the cornerstone of CMD management. Given underlying endothelial dysfunction and diffuse atherosclerosis, statins and angiotensin converting enzyme inhibitors are reasonable [72]. Indeed, intense risk factor modification with medications can dramatically improve myocardial ischemia [73]. Additionally, beta-blockers, calcium channel blockers, nitrates, and ranolazine are used to manage these patients who often have persistent symptoms, impacting their quality of life [74]. However, these drugs are not free of side effects and potential interactions. Additionally, considering comorbidities compounded with other medications to manage CMD, patient drug burden can be high. Thus, an adjunct strategy to treat CMD and reduce patient drug burden is needed. Lifestyle recommendations, including weight loss and aerobic exercise can independently improve CMD by improving CFR [75]. However, dietary interventions, particularly the consumption of polyphenol-rich plant-based foods, may also be a viable strategy to treat CMD in a targeted manner. 

### 4.1. Polyphenols

Polyphenols are secondary metabolites of plants with numerous bioactive properties (antioxidant, anti-inflammatory, anti-hypertensive, among others) and molecular targets [76]. Four major polyphenols classes exist, including flavonoids, phenolic acids, lignans and stilbenes (see Del Rio et al. [77] for an extensive review of polyphenol classes, bioavailability, structure, and metabolism). Berries are a particularly rich source of polyphenols compared to other fruits [78], and primarily contain phenolic acids as well as flavonoid including flavanols, flavonols and anthocyanins [79]. Table S1 highlights the polyphenolic profile of blueberries, strawberries, blackberries, red raspberries and cranberries, which are commonly consumed in the United States and Table S2 indicates their total polyphenol content [80]. Consumption of polyphenols, especially flavonoids, are associated with reduced CVD mortality [81,82,83]. To our knowledge, no human or animal studies currently exist which assess the effects of berries or their polyphenols in treating CMD. However, considering the high polyphenolic concentration of berries, it is likely that berries can be used to manage CMD in a pharmacological fashion by targeting the following pathways: (1) oxidative stress and inflammation, (2) RAAS and (3) β-adrenergic signaling. While these targets are of relevance across differing CVDs, they represent the primary sites of pharmacological intervention in CMD. Thus, their attenuation is of significant clinical relevance. This review focuses on commonly consumed berries: blueberries, strawberries, blackberries, cranberries and red and black raspberries, and their polyphenols (Table S1).

#### 4.1.1. Berry Polyphenols in Oxidative Stress and Inflammation

To counteract the detrimental effects of oxidative stress and inflammation, berry polyphenols may be of major therapeutic relevance in treating INOCA and CMD in a number of relevant preclinical models. For example, in spontaneously hypertensive rats (SHRs), gallic acid, a berry-derived phenolic acid, was provided in drinking water (1% concentration) for 16 weeks [84]. Cardiac protein expression of NOX2 was significantly reduced compared to SHR control animals, and this corresponded with decreased blood pressure. Further, hypertrophic fetal gene activation was significantly reduced with gallic acid, and cardiomyocyte hypertrophy was also significantly reduced. In a hyperglycemia-induced endothelial dysfunction model, isolated rat aorta were treated with 30 mM of glucose for 24 h in the presence or absence of 20 µM ellagic acid [85], a phenolic acid found in high concentrations in raspberries, strawberries and blackberries [86,87]. Ellagic acid was able to improve acetylcholine-induced vasodilation compared to hyperglycemic control aorta. Ellagic acid-treated aortas also had substantially reduced ROS production, NOX4 expression and reduced extracellular signal-regulated kinase (ERK)1/2 expression, a MAPK. 

In addition to NOX-mediated reductions in ROS, berry polyphenols can directly upregulate nuclear factor erythroid 2–related factor 2 (NRF2), a primary regulator of cellular antioxidant defenses [88]. In an ischemia-reperfusion model of cardiac injury, urolithin B, a metabolite of raspberry and strawberry [89,90], was provided to rats at a concentration of 0.7 mg/kg of body weight 48 and 24 h prior to ischemia-reperfusion injury [91]. Cardiac hemodynamics were substantially increased in the hearts of animals provided urolithin B, as was cardiac NRF2. Cardiac ROS production was also reduced and superoxide dismutase (SOD) was increased. While NRF2 genetic knockout blunted the beneficial effects of urolithin B, this is not true across berry polyphenols. For example, the blueberry and cranberry polyphenol myricetin [92] was provided to mice that underwent pressure overload-induced heart failure for six weeks at a dose of 200 mg/kg/d. Myricetin significantly reduced LVH as well as hemodynamic parameters of mouse hearts [93]. Myricetin also significantly increased cardiac NRF2 protein expression and decreased NF-κB activation. Interestingly, even with genetic NRF2 knockout, LVH was still reduced and hemodynamics were partially preserved. It was found that transforming growth factor beta-activated kinase 1 (TAK1), an upstream kinase of MAPK and NF-κB was inhibited with myricetin. Indeed, activation of NF-κB, as well as MAPKs: p38 and c-Jun N-terminal kinase, were significantly reduced. It can he hypothesized that significant oxidative stress was still present despite these changes, as siNrf2 transfected neonatal rat cardiomyocytes treated for 12 h with 50 μM phenylephrine, an inducer of hypertrophy, and 20 μM myricetin resulted in a significant increase in ROS as indicated by reduced SOD, catalase (catalyzes the conversion of H_2_O_2_ to H_2_O) and increased 4-hydroxynonenal (HNE; indicator of lipid peroxidation) protein expression despite reduced hypertrophy and fetal gene activation compared with phenylephrine alone [93]. Thus, berry polyphenols likely target every stage of oxidative stress and the inflammatory process. Of note, the investigations presented above are by no means exhaustive as berry polyphenols appear to mitigate inflammatory signaling and oxidative stress in a number of CVD models [94,95,96,97,98,99,100,101]. 

#### 4.1.2. Regulation of RAAS by Berry Polyphenols

Berry polyphenols likely possess pharmacological effects in a similar manner to medications which inhibit RAAS-related enzymes. For example, in human embryonic kidney (HEK) 293 cells, pretreatment with 10 μM dexamethasone significantly increased the activity of ACE; however, 100 μM of the flavonol quercetin and anthocyanins cyanidin and delphinidin, significantly decreased ACE activity regardless of dexamethasone pretreatment [102]. Although Captopril exerted these ACE inhibitory effects to a greater extent than these polyphenols, protein expression of ACE was not significantly decreased with either Captopril or quercetin, but it was decreased with cyanidin and delphinidin. While this in vitro study suggests that quercetin may be inhibiting the activity of ACE without changing its expression, these effects may not translate in vivo. Rats that received 10 mg/kg/day quercetin intraperitoneally for 14 days prior to receiving a single intravenous bolus of Ang I or Ang II in doses ranging from 0.03 to 10 μg/kg did not experience any reduction in blood pressure compared to control animals with Ang I or Ang II alone [103]. Plasma ACE activity was also not decreased due to quercetin supplementation. 

Components of RAAS are expressed throughout the cardiovascular system, including AGT, renin and ACE [104], all of which are potential targets of berry polyphenols. While little work has evaluated the effect of polyphenols on AGT expression, it is likely that reductions in inflammation would attenuate AGT expression in both the liver and kidney [105,106,107]. However, this effect is not clear in the heart, as cardiac-restricted TNF-α overexpression resulted in reduced cardiac AGT, but overexpression of ACE compared to wild-type littermates [108]. Regardless, cardiac Ang II was significantly increased in this transgenic line, suggesting that cardiac ACE and systemic components of RAAS may be of greater relevance than cardiac AGT, and that inflammation may drive their expression. In ECs, pretreatment with blueberry anthocyanins, malvidin, malvidin-3-glucoside, and malvidin-3-galactoside (5 μg/mL), for 4 h followed by 24 h of high-glucose conditions (30 mM) significantly decreased ACE protein expression compared with high-glucose alone [109]. Likewise, in vivo, consumption of a 3% blueberry diet by SHRs for two weeks resulted in a significant reduction in serum ACE activity compared to untreated animals [110]. Further, gallic acid-supplementation (1% in drinking water) for 24 weeks resulted in a significant reduction in both cardiac and aortic ACE and AT_1_R, which corresponded with decreased blood pressure and reduced aortic wall thickness compared to SHR controls [84]. Additionally, AT_1_R is a likely target of polyphenols, as AT_1_R was also reduced in this model [84]. An additional mechanism of decreased AT_1_R is mediated by sirtuin 1 (SIRT1) upregulation as observed in VSMCs treated with resveratrol, a SIRT1 activator [111]. While SIRT1 is classically known as a positive mediator of SOD2, NRF2 and eNOS [112], its expression is upregulated by a number of berry-derived polyphenols, including quercetin [113], myricetin [114] and delphinidin-3-glucoside [115]. 

Aldosterone may also be a target of berry polyphenols. For example, in adrenal glands isolated from SHRs, incubation of 500 μM quercetin or chlorogenic acid for 24 h resulted in a ~36% and ~33% reduction in aldosterone production compared to untreated glands [116]. Further, quercetin may also reduce aldosterone activity, as aldosterone-induced renal epithelial sodium channel activity was reduced in the presence of 100 μM of quercetin for 24 h [117]. Aldosterone also impacts ECs and may dimish endothelial function via the mineralocorticoid receptor (MR) by increasing NOX expression independent of blood pressure [118]. Preliminary evidence suggests that polyphenols, such as kaempferol, may also inhibit MR in human umbilical vein endothelial cells (HUVECs), resulting in the reduction of ROS and inflammatory signaling [119,120]. Thus, berries may target RAAS primarily via reduced ACE activity and decreased AT_1_R but may also inhibit the production and action of aldosterone.

#### 4.1.3. Berry Polyphenols as Adrenergic Receptor Inhibitors

Berries and their polyphenols may be an efficacious alternative to classical pharmaceutical adrenergic receptor blockers. For example, rats consumed an 8% blueberry diet for 13 weeks and VSMC-dependent aortic constriction was assessed with endothelium-denuded aortic rings and whole intact rings [121]. Phenylephrine-induced vasoconstriction was significantly blunted in whole intact aortic rings from animal which consumed the blueberry diet. However, the vasorelaxation effects of blueberry in denuded aortic rings were not apparent, suggesting that blueberries mediate their α1AR inhibitory effects primarily via the endothelium. This is further evidenced in aortic rings isolated from SHRs fed an 8% blueberry diet for eight weeks [122]. While blueberry reduced phenylephrine-induced vasoconstriction as predicted compared to control, the addition of l-N^G^-monomethyl arginine (L-NMMA), a NOS inhibitor, significantly blunted these effects in the blueberry group, suggesting a clear role of the endothelium in mediating the adrenergic response. These findings are consistent across other preclinical models, including obesity- and high-fat diet-induced vasoconstriction [123,124].

In the heart, berries and their polyphenols may also function as adrenergic receptor blockers. In rat cardiomyocytes pretreated with 6.55 µg/mL of blueberry-derived flavonoids and anthocyanins, 0.25 µM norepinephrine stimulation for 24 h, an adrenergic receptor agonist, resulted in a significant attenuation of aberrant contractility compared to norepinephrine alone [125]. Further, norepinephrine resulted in a significant increase in cardiomyocyte hypertrophy, apoptosis, and ROS, all of which were attenuated with blueberry polyphenols. In vivo, rats were pretreated with 200 mg/kg/d of malvidin-3-glucoside, a berry anthocyanin, for 21 days followed by HF induction with isoproterenol (85 mg/kg/d for the last 2 days), a βAR agonist. Malvidin-3-glucoside significantly attenuated cardiac injury as evidenced by dramatically reduced lactate dehydrogenase and creatine kinase, products of cardiomyocyte lysis. Further, isoproterenol alone resulted in a significant increase in cardiac NF-κB signaling, pro-inflammatory cytokine release, and a dramatic decline in catalase, SOD, and reduced glutathione, all of which were reversed by malvidin-3-glucoside [126]. These βAR-antagonistic effects are consistent with other polyphenols in isoproterenol-induced HF, as observed with gallic acid [127], quercetin [128] and resveratrol [129].

## 5. Clinical Effects of Berries: Implications in CMD

Combined, the effects of berries and their polyphenols in reducing inflammation, oxidative stress, RAAS, and adrenergic signaling in preclinical trials suggests that berries may be efficacious in treating CMD (Table 1). However, no human studies currently exist which assess this hypothesis. Nonetheless, a number of human studies exist utilizing berries in the treatment of CVDs or CMD-relevant physiological and biochemical risk factors. Discussed below are a sampling of clinical studies which assess the efficacy of commonly consumed berries, namely blueberries, strawberries, cranberries, red and black raspberries, in CMD-relevant measures. Blackberries were not discussed below due to a paucity of clinical data.

### 5.1. Blueberries

Smoking is a known risk-factor for CMD due to increased systemic oxidative stress and inflammation [130], and even light smoking can lead to CFR reduction [131]. In an acute smoking study, subjects consumed 300 g of fresh blueberry drink or placebo and then smoked one cigarette [132]. Impaired microvascular function, as assessed by reactive hyperemia (RHI), as well as arterial stiffness, as assessed by the augmentation index (AIx) were significantly exacerbated due to smoking. However, RHI reductions were attenuated with blueberry consumption. Further, systolic blood pressure elevations induced by smoking were decreased with blueberry consumption. A reduction in oxidative stress is a reasonable hypothesis, since 25 g of freeze-dried wild blueberry powder for six weeks in subjects with at least one CVD risk factor resulted in a reduction of H_2_O_2_-induced DNA damage [133]. Further, in healthy human subjects, postprandial consumption of ~1.5 cups of wild blueberry significantly improved endothelial function and reduced neutrophil NOX expression, which tightly corresponded with hourly serum polyphenol fluctuations [134], suggesting a clear role for blueberries in mediating oxidative stress. However, this was not observed in a similarly designed smoking study by Del Bo’ et al. [135]. It has been observed that smoking results in a rapid increase in serum ACE [136], thus, it is possible that blueberries are attenuating RAAS in this smoking model resulting in reduced blood pressure and arterial stiffness, although this was not directly evaluated. 

Diabetics have also been observed to have impaired CFR [137] and are likely to have CMD due to impaired endothelial function exacerbated by oxidative stress and inflammation [138]. In middle-aged obese subjects with type II diabetes, the consumption of 1 cup of blueberries for six months improved AIx and significantly improved flow-mediated dilation (FMD) [139]. In obese subjects with metabolic syndrome, 45 g of freeze-dried blueberry powder improved RHI after six weeks [140]. While blood pressure was not decreased, in a similar investigation which utilized 50 g of freeze-dried blueberries for eight weeks, both systolic and diastolic blood pressure decreased (−6% and −4%, respectively) significantly more than in control subjects [141]. Reductions in plasma oxidized LDL and serum malondialdehyde (MDA) and HNE concentrations, proxies for systemic oxidative stress, were also greater in blueberry-supplemented subjects. Additionally, in subjects with metabolic syndrome, six-week consumption of 45 g of freeze-dried blueberries resulted in a reduction of both serum and monocytic ROS derived from O_2_^•−^, H_2_O_2_ and hydroxyl radical (^•^OH), in addition to a decrease in TNF-α and IL-6 mRNA expression [142]. Reductions in oxidative stress would presumably result in increased NO bioavailability, likely contributing to the observed improvements in vascular stiffness and function. Indeed, in two separate investigations, the consumption of 240 mL of wild blueberry juice for seven days [143] or 22 g of freeze-dried blueberry powder for eight weeks [144] significantly increased serum NO in diabetics and pre-hypertensive woman, respectively. 

### 5.2. Strawberries

Strawberries also appear to be efficacious in managing CMD-associated measures of interest, albeit to a lesser extent compared with blueberries. In hyperlipidemic subjects, 110 g/d for six weeks resulted in a reduction of oxidized LDL compared to placebo when challenged with a high-fat meal [145]. In obese subjects, 25 g/d of freeze-dried strawberries for 12 weeks resulted in a significant reduction in serum MDA and HNE concentrations [146]. Despite these findings, weak effects are observed with regard to vascular function. In overweight adolescent males, two-week consumption of 50 g freeze-dried strawberry powder increased serum NO metabolite concentrations despite no changes in RHI [147]. Similarly, in pre-hypertensive woman, 50 g/d of freeze-dried strawberry for eight weeks did not reduce arterial stiffness despite a significant increase in NO metabolites [148]. However, 25 g/d of freeze-dried strawberry did reduce arterial stiffness and decrease systolic blood pressure compared to baseline levels despite no significant changes in NO [148], suggesting other protective mechanisms at play. 

### 5.3. Cranberries

A number of clinical investigations have demonstrated that cranberries are quite efficacious in improving vascular function. For example, young healthy males consumed 450 mL of either 25, 48, 76, 94, or 117% concentrated cranberry juice equivalent to 409, 787, 1238, 1534, and 1910 mg of total polyphenols, respectively [149]. While all doses of cranberry were effective in improving FMD postprandially, 76% cranberry juice appeared most efficacious. Interestingly, time-dependent changes were observed, with peak FMD at 4 h which tightly corresponded with changes in polyphenols in serum. However, 117% cranberry juice concentrate demonstrated the greatest changes in polyphenols despite less dramatic changes in FMD compared with the 76% cranberry juice concentrate. Acute changes in FMD have also been observed with 450 mL of 54% juice in patients with coronary artery disease [150]. However, chronic supplementation appears less effective, as two weeks of 450 mL/d of 54% cranberry juice did not result in improvements in FMD [150], which was similar to observations in overweight men consuming 500 mL/d of 27% cranberry juice for four weeks, in which AIx was unchanged [151]. Nonetheless, chronic supplementation with 480 mL/d of 27% cranberry juice for eight weeks in women with metabolic syndrome resulted in a significant increase in plasma antioxidant capacity and decreased oxidized LDL as well as MDA and HNE despite no changes in inflammatory biomarkers [152]. In overweight, middle aged subjects, 480 mL of ocean spray cranberry juice (% cranberry unspecified) for eight weeks significantly reduced diastolic blood pressure and CRP, a measure of systemic inflammation [153]. Eight weeks of 500 mL/d of cranberry juice consumption also decreased oxidized LDL and endothelial inflammatory biomarkers, intracellular adhesion molecule-1 (ICAM-1) and vascular cell adhesion molecule-1 (VCAM-1) in overweight, middle-aged subjects [154]. Thus, cranberries appear to have protective vascular effects and may decrease oxidative stress and inflammation.

### 5.4. Red and Black Raspberries

As with blueberries and cranberries, red raspberries appear to have potent postprandial effects on FMD. For example, 200 and 400 g of fresh red raspberries in healthy subjects resulted in a significant increase in FMD at both 2 and 24 h postprandially, which corresponded with plasma changes in ellagic acid and urolithin metabolites, respectively [90]. In type II diabetics, the consumption of a high-fat meal with 250 g of freeze-dried raspberries resulted in significantly lower postprandial concentration of TNF-α and IL-6 compared to control [155]. These protective effects were similar after four weeks of consuming 250 g/d of fresh raspberries, in which fasting TNF-α and IL-6 were lower than control, as was systolic blood pressure. Interestingly, in overweight and obese subjects at risk for metabolic syndrome, 280 g/d of frozen red raspberry for eight weeks resulted in transcriptional changes of cells from whole blood, with downregulation of genes associated with inflammatory cytokine production [156].

Black raspberries also have protective vascular effects. For example, 750 mg of dried black raspberry powder were provided to subjects with metabolic syndrome for 12 weeks [157]. Significant reductions in TNF-α, IL-6 and ICAM-1 were observed compared to placebo, which corresponded with a significant reduction in AIx. Additionally, in patients with metabolic syndrome, 750 mg/d of black raspberry extract powder for 12 weeks significantly improved FMD compared to placebo [158]. Lastly, in subject with pre-hypertension, 1500 or 2500 mg/d of black raspberry extract powder for eight weeks significantly reduced 24 h systolic blood pressure, as well as serum IL-6 and TNF-α in a dose-dependent manner [159]. Interestingly, serum renin and ACE appeared to decrease in subjects consuming the 2500 mg/d of the black raspberry powder; however, this difference was not statistically significant between groups. 

## 6. Limitations

Available clinical evidence suggests that berries, particularly blueberries, cranberries, red raspberries and black raspberries, elicit protective effects on vascular function, potentially improving CMD. However, a major limitation of this conclusion is that no clinical studies exist utilizing berries or their polyphenols to treat CMD. Rather, clinical and pre-clinical evidence suggests that the polyphenols found in berries target the underlying drivers of CMD in a more comprehensive fashion compared to current pharmacologics. Specific recommendations regarding which berry to consume for CMD or which polyphenol is most efficacious cannot be appropriately made, and any such recommendation would be unfounded based on the current state of the literature. Investigations utilizing berries and their polyphenols in treating CMD are needed to determine efficacy and dosage.

## 7. Conclusions

INOCA is a clinically significant problem and is associated with morbidity and mortality that is comparable to obstructive CAD. CMD is a major contributor of adverse outcomes in INOCA. The underlying drivers of CMD, namely, oxidative stress and inflammation, upregulated RAAS, and adrenergic signaling, represent major therapeutic targets. While medications attempt to address these pathways, polypharmacy is typically not desirable and burdens patients with side effects and potential drug interactions. Compelling preclinical evidence suggests that berry-derived polyphenols can directly inhibit αAR, βAR, ACE, aldosterone, AT_1_R, NF-κB, MAPK, NOX and increase NRF2, thus targeting major pathways of interest in CMD that pharmaceuticals either partially address or do not address at all. Further, a number of clinical studies demonstrate the efficacy of berries in improving macro- and microvascular function, with blueberries, cranberries, and red and black raspberries being particularly efficacious, with less compelling evidence for strawberries. Because no clinical studies utilizing berries currently exist that have aimed to treat CMD, future clinical trials should utilize berries clinically as an adjunct treatment of this disease.

## Figures and Tables

**Table 1:** Potential Therapeutic Targets of Polyphenols in Coronary Microvascular Dysfunction.

Cellular Target	Berry/Polyphenol(s)	Cellular Effect and Physiological Consequence
αAR ↓ [121,122,123,124]	Blueberries	Decreased phenylephrine-induced αAR signaling in the vasculature with reduced VSMC-mediated vasoconstriction. Thus, epicardial microvascular blood flow can potentially be improved in the presence of classical αAR agonists, epinephrine, and norepinephrine.
βAR ↓ [125,126,127,128,129]	Blueberries, malvidin, gallic acid, quercetin, resveratrol and syringic acid.	Decreased isoproterenol-, epinephrine- and norepinephrine-induced βAR signaling in the heart, reducing LVH, aberrant Ca^2+^ handling, and cardiac ROS. These cumulative effects preserve myocardial architecture, thus, maintaining adequate microvascular flow.
ACE ↓ [84,109,110]	Gallic acid and blueberries	Decreased ACE expression throughout the cardiovascular system, reducing the cleavage of Ang I to Ang II and reducing hypertension. ACE inhibitors are classically used in CMD treatment; thus, berry polyphenols may target ACE in a pharmacological fashion.
Aldosterone ↓ [116,117,119,120]	Caffeic acid, quercetin and kaempferol	May decrease the synthesis of aldosterone in the adrenal cortex and decrease the action of aldosterone in the kidneys, resulting in reduced blood pressure, decreasing microcirculatory resistance. Additionally, polyphenols may decrease MR activity in the endothelium, resulting in reduced endothelial dysfunction.
AT_1_R ↓ [84,111]	Gallic acid and resveratrol	Decreased AT_1_R expression in the heart and endothelium, reducing downstream AT_1_R signaling, potentially preserving endothelial function, and reducing LVH. Angiotensin receptor blockers are common medications prescribed to patients with CMD, thus, polyphenols may act in a similar pharmacological fashion.
NF-κB ↓ [93,94,95,96,97,98,99]	Myricetin, resveratrol, cyandin-3-glucoside, quercetin and catechin	Decreased NF-κB phosphorylation and nuclear translocation leading to a decrease in inflammatory cytokine expression in ECs, VSMCs, and cardiomyocytes. In the endothelium, this leads to a decrease in leukocyte infiltration, decreasing LDL phagocytosis and diffuse atherosclerosis in the sub endothelial space.
MAPK ↓ [85,93,98,100]	Blackberry, raspberry and black raspberry polyphenol extracts, ellagic acid, myricetin, quercetin and catechin	Polyphenols decrease cardiomyocyte MAPK signaling, reducing fetal gene activation and a subsequent attenuation of cardiomyocyte hypertrophic growth. Decreased myocardial hypertrophy prevents an impediment of lumenal space of arteriole and pre-arterioles of epicardial microvasculature.
NOX ↓ [84,85,100,101]	Blackberry, raspberry and black raspberry polyphenol extracts, gallic acid, cyanidin-3-glucoside	Decreased NOX protein expression in ECs, VSMCs, and cardiomyocytes, which reduces ROS, thereby reducing redox sensitive kinases upstream from MAPK and NF-κB. In ECs, reduced ROS from NOX prevents eNOS uncoupling and increases NO bioavailability. In VSMCs, reduced NOX-derived ROS prevent aberrant intracellular Ca^2+^ fluctuations, thus, endothelial-independent and -dependent dysfunction is attenuated.
NRF2 ↑ [91,93,95,96]	Urolithin, B, myricetin and cyanidin-3-glucoside	Increased nuclear translocation of NRF2, increasing transcription of antioxidant enzymes, leading to the neutralization of ROS. Thus, upregulated NRF2 can lead to quenching of excessive ROS produced from NOX and other potential ROS sources.

Abbreviations: αAR, α-adrenergic receptor; βAR, β-adrenergic receptor; ACE, angiotensin converting enzyme; AT1R, angiotensin II type 1 receptor; Ca^2+^, calcium; ECs, endothelial cells; LVH, left ventricular hypertrophy; LDL, low density lipoprotein; MAPK, mitogen-activated protein kinase; MR, mineralocorticoid receptor; NOX, NADPH-oxidase; NRF2, nuclear factor erythroid 2-related factor 2; NF-κB, nuclear factor kappa-light-chain-enhancer of activated B cells; NO, nitric oxide; ROS, reactive oxygen species; VSMCs, vascular smooth muscle cells.

## Supplementary Materials

Supplementary materials can be found at https://www.mdpi.com/1422-0067/22/7/3373/s1.
