# Supplementary material for: Berries and Their Polyphenols as a Potential Therapy for Coronary Microvascular Dysfunction: A Mini-Review
Source: Int J Mol Sci. 2021 Mar 25;22(7):3373. doi: 10.3390/ijms22073373 (PMC8036956; doi:10.3390/ijms22073373)
Supplement: Supplementary file 1 [file ijms-22-03373-s001.pdf]

Table S1. Flavonoid and phenolic acid profile of berries commonly consumed in the United States [81].

	Blueberry	Strawberry	Blackberry	Red Raspberry	Black Raspberry	Cranberry
**ANTHOCYANINS**						
Cyanidin		X		X		
Cyanidin 3-O-(6''-acetyl-galactoside)	X					
Cyanidin 3-O-(6''-acetyl-glucoside)	X					
Cyanidin 3-O-(6''-dioxalyl-glucoside)	X		X			
Cyanidin 3-O-(6''-malonyl-glucoside)			X			
Cyanidin 3-O-(6''-succinyl-glucoside)		X				
Cyanidin 3-O-arabinoside	X					X
Cyanidin 3-O-galactoside	X					X
Cyanidin 3-O-glucoside	X	X	X	X	X	X
Cyanidin 3-O-glucosyl-rutinoside				X		
Cyanidin 3-O-rutinoside			X	X		
Cyanidin 3-O-sophoroside				X		
Cyanidin 3-O-xyloside			X			
Delphinidin 3-O-(6''-acetyl-galactoside)	X					
Delphinidin 3-O-(6''-acetyl-glucoside)	X					
Delphinidin 3-O-arabinoside	X					
Delphinidin 3-O-galactoside	X					
Delphinidin 3-O-glucoside	X			X		
Malvidin 3-O-(6''-acetyl-galactoside)	X					
Malvidin 3-O-(6''-acetyl-glucoside)	X					
Malvidin 3-O-arabinoside	X					
Malvidin 3-O-galactoside	X					
Malvidin 3-O-glucoside	X			X		
Pelargonidin		X				
Pelargonidin 3-O-(6''-malonyl-glucoside)		X				
Pelargonidin 3-O-(6''-succinyl-glucoside)		X				
Pelargonidin 3-O-arabinoside		X				
Pelargonidin 3-O-glucoside		X		X		
Pelargonidin 3-O-glucosyl-rutinoside				X		
Pelargonidin 3-O-rutinoside		X		X		
Pelargonidin 3-O-sophoroside				X		
Peonidin 3-O-(6''-acetyl-galactoside)	X					
Peonidin 3-O-(6''-acetyl-glucoside)	X					
Peonidin 3-O-galactoside	X					X
Peonidin 3-O-glucoside	X					X
Petunidin 3-O-(6''-acetyl-galactoside)	X					

Petunidin 3-O-(6''-acetyl-glucoside)	X					
Petunidin 3-O-arabinoside	X					
Petunidin 3-O-galactoside	X					
Petunidin 3-O-glucoside	X					
**FLAVANOLS**						
(+)-Catechin		X	X	X		
(+)-Gallocatechin		X				
(-)-Epicatechin		X	X	X		
(-)-Epicatechin 3-O-gallate		X				
(-)-Epigallocatechin		X	X	X	X	
Procyanidin polymers		X	X	X		
**FLAVONOLS**						
Kaempferol 3-O-glucoside		X		X		X
Kaempferol 3-O-glucuronide		X				
Morin		X				
Myricetin	X		X			
Myricetin 3-O-arabinoside						X
Quercetin			X	X	X	
Quercetin 3-O-arabinoside						X
Quercetin 3-O-galactoside			X			X
Quercetin 3-O-glucuronide		X		X		
Quercetin 3-O-glucoside			X	X		
Quercetin 3-O-glucosyl-xyloside			X			
Quercetin 3-O-rhamnoside						X
Quercetin 3-O-rutinoside			X	X	X	
Quercetin 3-O-xyloside			X			
Quercetin 3-O-xylosyl-glucuronide			X			
**HYDROXYBENZOIC ACIDS**						
2,4-Dihydroxybenzoic acid						X
3-Hydroxybenzoic acid						X
4-Hydroxybenzoic acid 4-O-glucoside		X	X			X
5-O-Galloylquinic acid		X				
Benzoic acid						
Ellagic acid		X	X	X		
Ellagic acid acetyl-arabinoside				X		
Ellagic acid acetyl-xyloside				X		
Ellagic acid arabinoside				X		
Ellagic acid glucoside		X				
Gallic acid			X	X	X	

Protocatechuic acid 4-O-glucoside			X			
Galloyl glucose			X			
Lambertianin C				X		
Sanguiin H-6				X		
Vanillic acid						X
**HYDROXYCINNAMIC ACIDS**						
3-caffeoylquinic acid			X	X	X	
3-feruloylquinic acid			X			
3-p-coumaroylquinic acid			X			
4-caffeoylquinic acid			X	X	X	
5-caffeoylquinic acid	X	X	X	X	X	
Caffeic acid						X
Caffeoyl glucose		X	X			
Cinnamic acid		X				X
Feruloyl glucose		X	X			
Ferulic acid					X	X
P-coumaric acid		X	X	X	X	X
P-coumaric acid 4-o-glucoside		X	X	X		
P-coumaroyl glucose		X	X			

Table S2. Total polyphenol content (TPC) of commonly consumed berries [81].

Berry	TPC (mg/100g)
Blueberries (Highbush), raw (*Vaccinium corymbosum L.*)	223.41
Blackberries, raw (*Rubus L.*)	569.43
Black Raspberries, raw (*Rubus occidentalis L.)*	980.00
Cranberries, raw (*Vaccinium macrocarpon Aiton*)	315.00
Red raspberries, raw (*Rubus idaeus L.*)	154.65
Strawberries, raw (*Fragaria L.*)	289.20
